# Supplementary figures and images for: Parallel adaptation and admixture drive the evolution of virulence in the grapevine downy mildew pathogen
Source: PLoS Pathog. 2026 Mar 10;22(3):e1014041. doi: 10.1371/journal.ppat.1014041 (PMC13048484; doi:10.1371/journal.ppat.1014041)

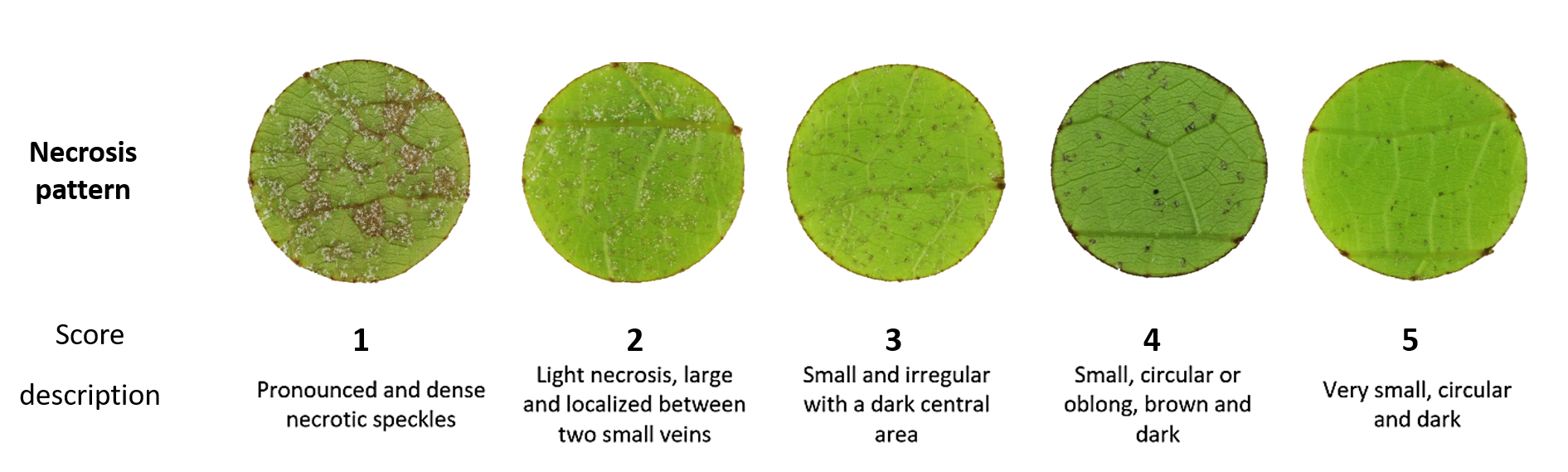

Supplement: S1 Fig — Necrosis patterns on leaf discs were evaluated at 6 dpi by assessing their size, shape and color. Higher necrosis scores correspond to more efficient immune responses. Photos were taken by the authors. Scores were attributed based on the scale proposed by Paineau et al. [35]. (TIFF) [file ppat.1014041.s001.tiff]

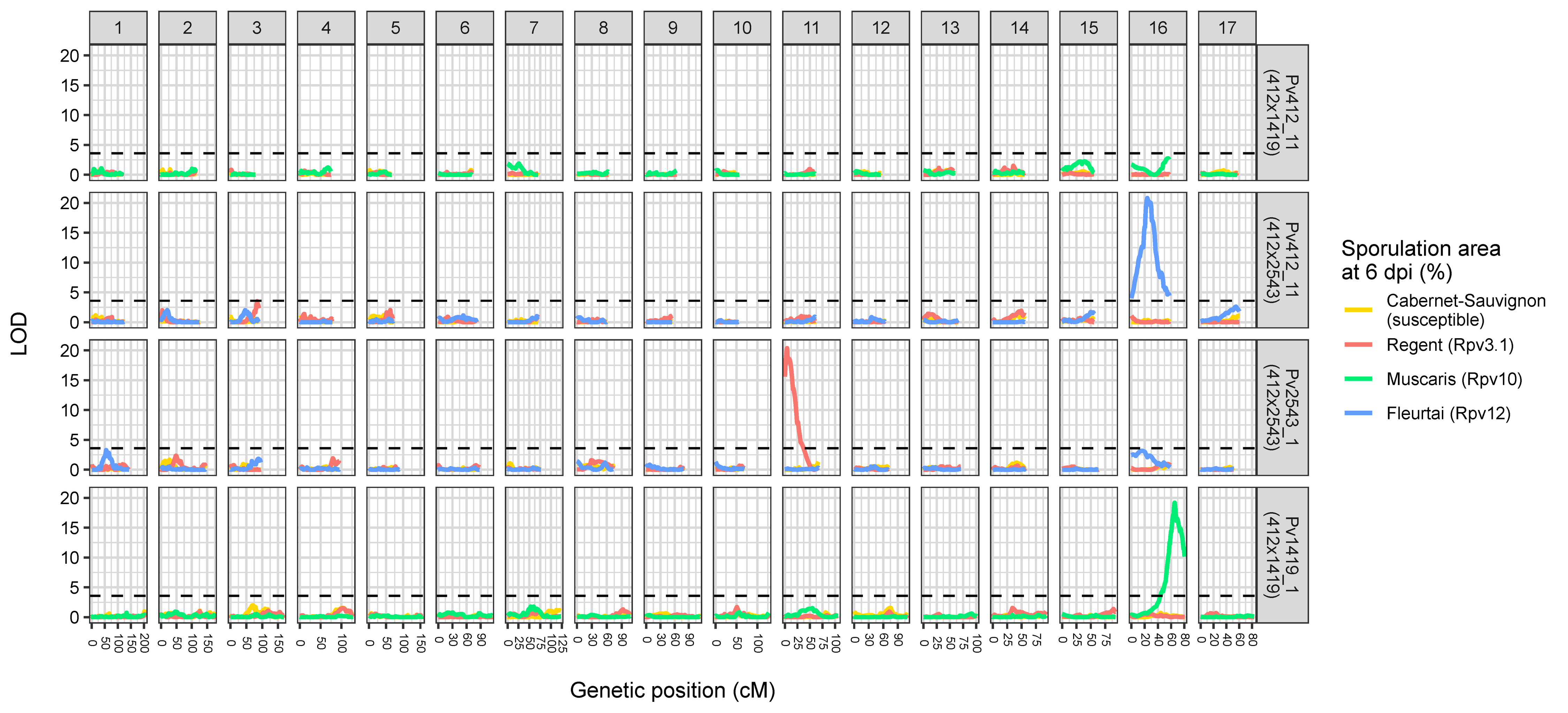

Supplement: S2 Fig — One map was obtained for each parent of each cross. The detection of a QTL in one of the parental maps indicate a phenotypic difference in the progeny depending on which marker alleles were transmitted by the parent. The black dashed line indicates a LOD significance level of 3.2 which was the lowest threshold value determined across the different QTL mappings (α = 0.05). LOD values were computed based on the percentage of sporulation area at 6 dpi. (TIF) [file ppat.1014041.s002.tif]

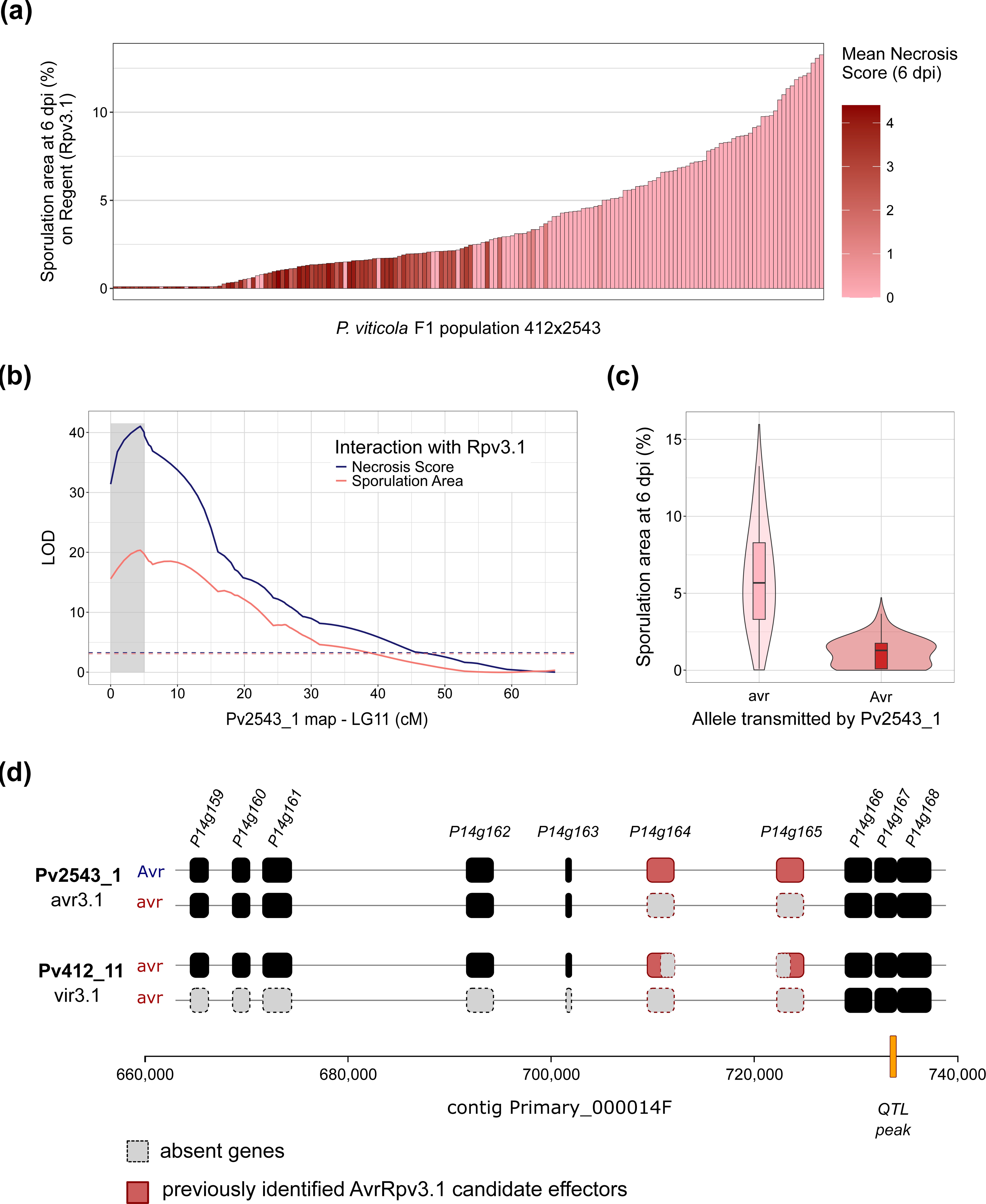

Supplement: S3 Fig — (a) Phenotypes distribution in the 412x2543 F1 progeny (N = 162) on cv. ‘Regent’ (Rpv3.1). (b) QTL mapping of Rpv3.1-breakdown in the linkage map of the avirulent parent Pv2543_1. The gray area indicates the credible interval of the QTL. Dashed lines indicate the LOD significance thresholds determined using 1000 permutations. Results on other linkage groups and other cultivars are available in S2 Fig. (c) Distribution of the sporulation area on Rpv3.1 depending on the inherited allele at the QTL. Horizontal lines in the boxplots signal the 25th, 50th and 75th percentiles. (d) Allelic configurations of the parent strains in the QTL, which corresponds to the same region previously identified by GWAS [36]. The marker corresponding to the peak of the QTL in the present study is indicated by an orange bar on the scale. The allele associated with avirulence corresponds to the non-deleted Pv2543_1 haplotype (named Avr on the left). The secreted proteins P14g164 and P14g165 (colored in red) are totally or partially deleted in the virulent haplotypes. (TIF) [file ppat.1014041.s003.tif]

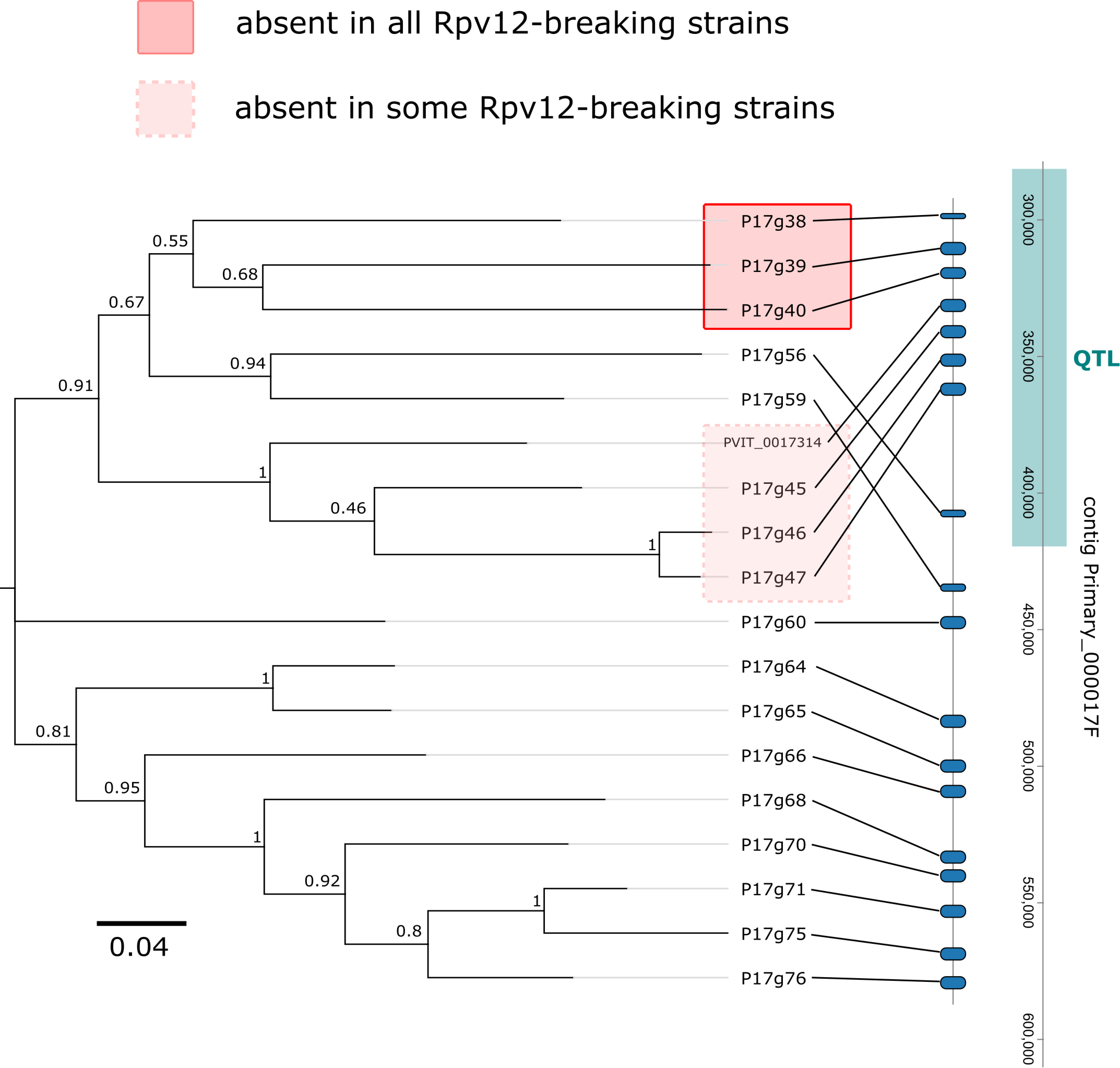

Supplement: S4 Fig — Bootstrap support values obtained from 1000 replicates are indicated for each node. RXLR genes absent in all or some Rpv12-breaking strains are highlighted in red boxes. The credible interval of the AvrRpv12 QTL on contig Primary_000017F is indicated in turquoise. (TIF) [file ppat.1014041.s004.tif]

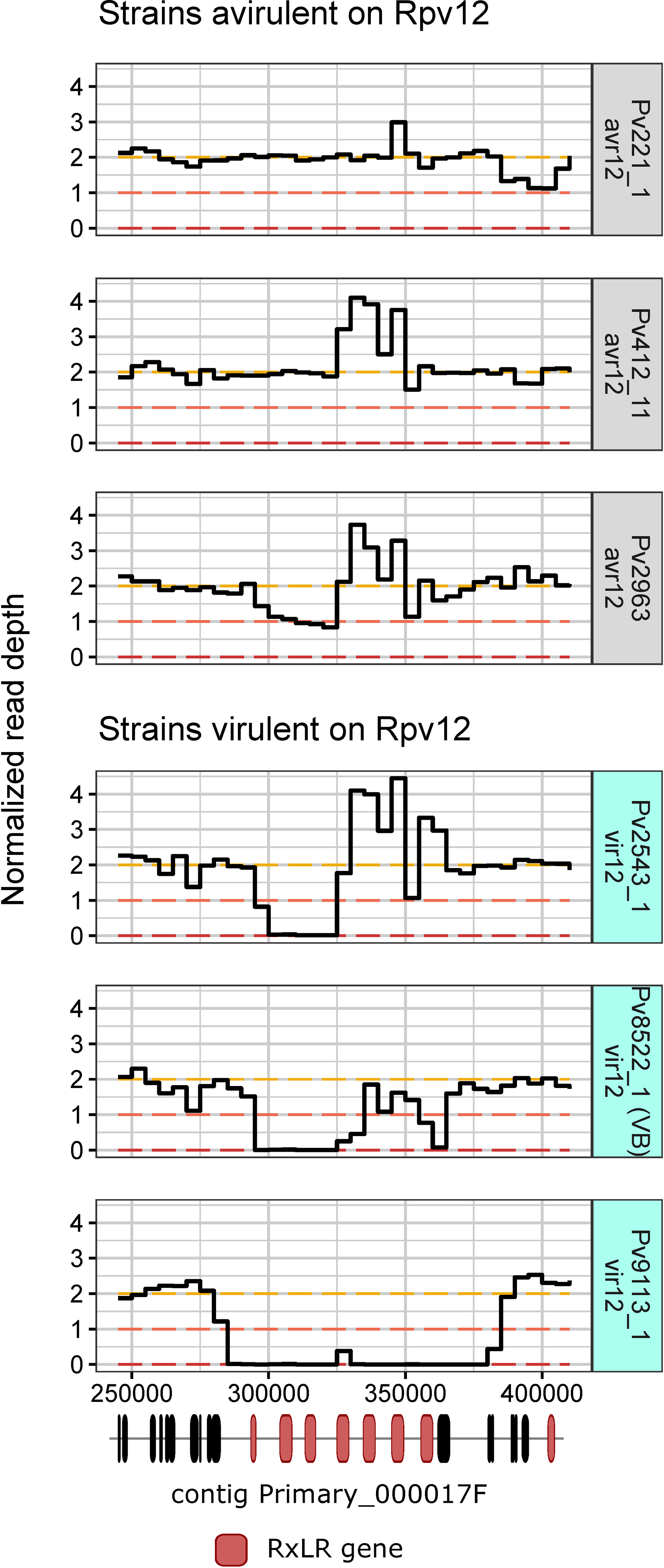

Supplement: S5 Fig — Copy number is indicated on the y-axis and calculated along 5 kb windows. At the bottom, coding sequences are indicated in black, or in red for RXLR genes. The reference avirulent strain Pv221_1 is at the top. Blue boxes signal strains virulent on Rpv12. Some strains present higher coverage for the fourth and fifth RXLR genes, suggesting they possess additional copies. Note the hemizygous profile of strain Pv2963, which was collected from the same plot as Pv2543_1 but is avirulent. (TIF) [file ppat.1014041.s005.tif]

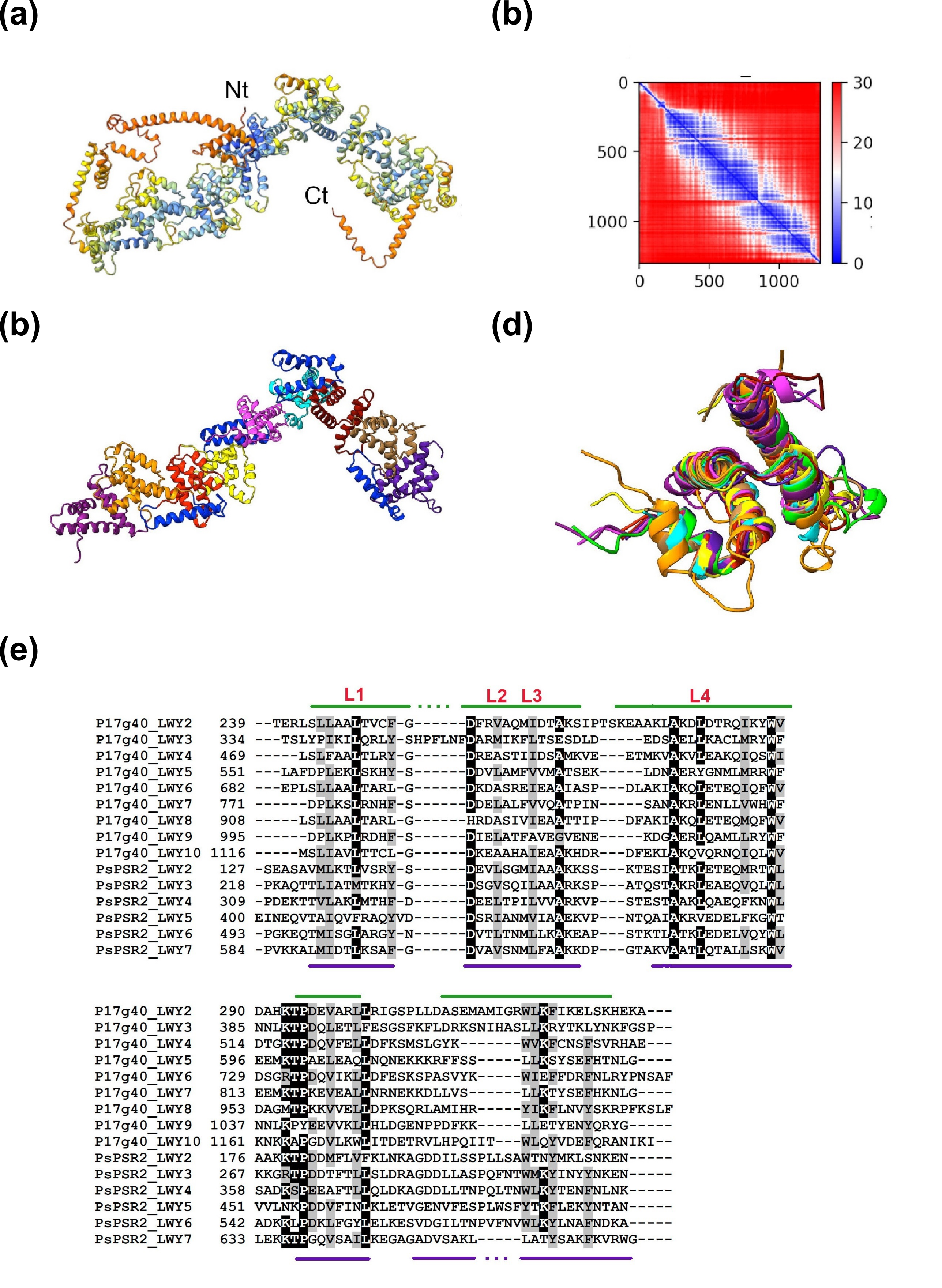

Supplement: S6 Fig — Candidate protein P17g40 code for an RXLR protein composed of several modules of the LWY domain. (a) Complete AlphaFold-predicted structure colored by predicted local distance difference test (pLDDT) (red: low confidence, blue: high confidence). The N terminus and C terminus of the molecule are indicated by ‘Nter’ and ‘Cter’. (b) Predicted aligned error (pAE) of the relative position of residues along the protein sequence. (c) Predicted structures with the 9 complete LWY modules highlighted in different colors. Sequences linking the different modules are shown in blue. Poorly predicted N- and C-terminal parts were trimmed for visual clarity. (d) Superimposition of all LWY domains of P17g40, using the third domain from the oomycete effector PsPSR2 as a reference. (e) Alignment of the P17g40 and the PsPSR2 sequences, skipping the first module that is shorter than the others in both proteins [26]. Green lines at the top of the alignment indicate alpha-helices sequences for P17g40, and purple lines at the bottom indicate those of PsPSR2. Conserved leucine residues contributing to the fold are highlighted in red. Black background indicate identity and gray background similarity (70% cutoff). (TIFF) [file ppat.1014041.s006.tiff]

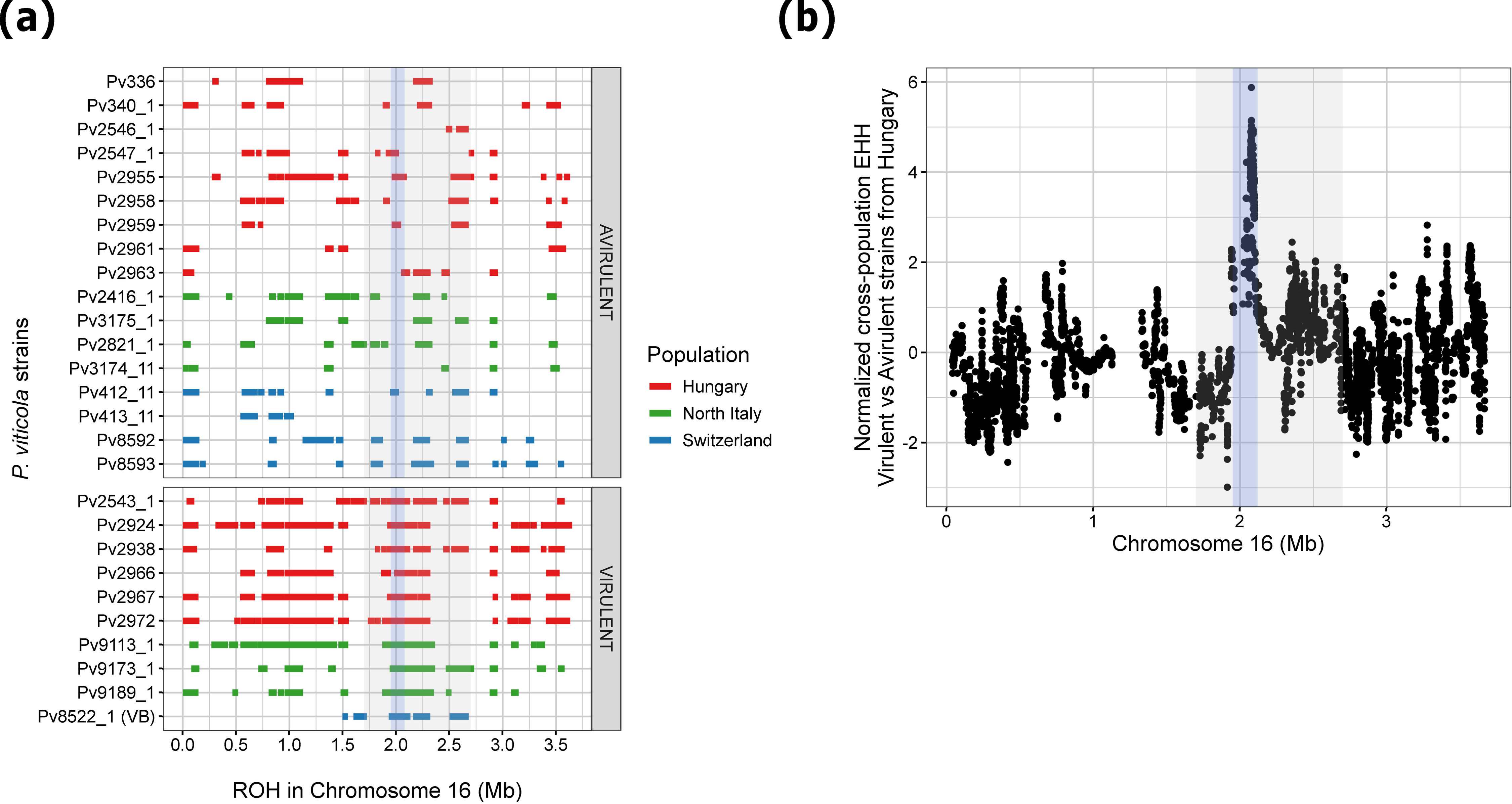

Supplement: S7 Fig — Blue boxes indicate the AvrRpv12 QTL and grey boxes show the limits of contig Primary_000017F. (a) Runs of Homozygosity (ROH) on chromosome 16. Lines indicate uninterrupted homozygous segments. Avirulent and virulent strains from three different geographical origins are shown. The VB strain was previously studied in Wingerter et al. [18]. Plot made using r/detectRUNS. (b) Cross-population Extended Haplotype Homozygosity (XP-EHH) calculated by Selscan along chromosome 16. Virulent strains from Hungary (n = 6) were compared to avirulent strains from the same country (n = 9). The highest positive scores are observed around AvrRpv12, suggesting positive selection in the virulent subpopulation. (TIF) [file ppat.1014041.s007.tif]

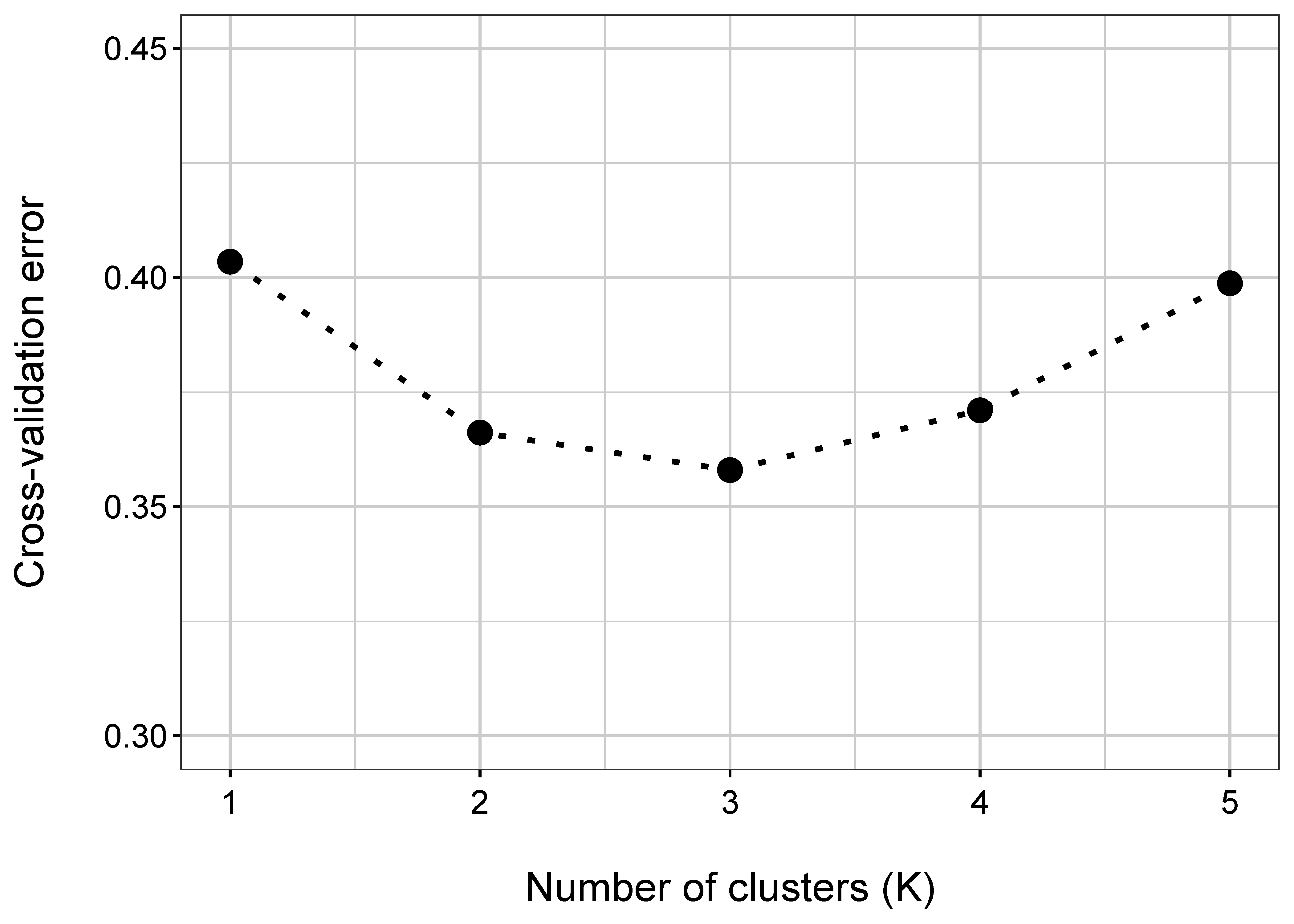

Supplement: S8 Fig — The value is lowest for K = 3, making it the best-fitting number of clusters. (TIF) [file ppat.1014041.s008.tif]
