# Supplementary material for: Parallel adaptation and admixture drive the evolution of virulence in the grapevine downy mildew pathogen
Source: PLoS Pathog. 2026 Mar 10;22(3):e1014041. doi: 10.1371/journal.ppat.1014041 (PMC13048484; doi:10.1371/journal.ppat.1014041)
Supplement: S1 Methods — (DOCX) [file ppat.1014041.s009.docx]

**Supplementary Methods for “Parallel adaptation and admixture drive the evolution of virulence in the grapevine downy mildew pathogen”**

Etienne Dvorak, Thomas Dumartinet, Isabelle D. Mazet, Alexandre Châtaigner, Manon Paineau, Dario Cantú, Pere Mestre, Marie Foulongne-Oriol, François Delmotte

**Methods S1** Chromosome-level assembly of strain Pv1419 1, quality assessment and annotation

HiFi reads of Pv1419 1 were assembled using a two-step procedure combining HiFiasm v0.16.1-r37412 (Cheng et al, 2021) and the HaploSync tool suite v1.0 (Minio et al, 2022). To generate the most contiguous and high-quality draft genome assembly and determine the optimal settings in HiFiasm, multiple parameter combinations were tested, generating a total of 252 diploid assemblies. All assemblies were evaluated for haplotype balance, length and overall quality. Each haplotype was expected to have a cumulative length between 100 and 115 Mbp, with a difference of less than 10 Mbp between the two haplotypes. Assembly fragmentation was assessed based on contig length distribution, with high-quality assemblies characterized by a longest contig exceeding 7.5 Mbp and a high proportion of large contigs. To assess assembly completeness and uniformity, NG-values (NG0–NG99) were calculated for both haplotypes using an expected genome size of 115 Mbp (Voglmayr and Greilhuber, 1998). The full range of NG-values was compared between haplotypes to verify their homogeneity, ensuring consistent contiguity and completeness across the genome and avoiding cases where one haplotype displayed substantially higher NG-values than the other. Based on these descriptive statistics, the ten most promising assemblies were screened for contamination. To remove contaminants reads, genetic markers developed in Dvorak et al (2025) were used to identify contigs belonging to the *P. viticola* genome. Contigs lacking these markers were aligned against the RefSeq genome (O’Leary et al, 2016) and classified using MEGAN6 (Huson et al, 2016) to identify contigs within the oomycete class. Verified oomycete contigs were incorporated into the *P. viticola* assembly, and unassigned contigs were also retained.

HiFi reads corresponding to contaminant contigs were removed, and HiFiasm was rerun using the cleaned dataset. The resulting assemblies were evaluated according to the quality metrics described above. Genetic markers were then mapped onto the best assemblies to assess completeness and guide the final selection. The assembly generated with parameters a = 2, k = 61, w = 71, f = 25, r = 5, s = 0.6, D = 10, N = 75, n = 10, and m = 500,000 showed the highest completeness and overall quality and was selected for the second step of the assembly. Next, scaffolded, haplotype-resolved, chromosome-scale pseudomolecules were constructed for both haplotypes using the *P. viticola* genetic map (Dvorak et al, 2025) and HaploSync tool suite v1.0 (Table S1). During this second step, two iterations of HaploSplit and two iterations of HaploFill were performed to reduce fragmentation and finalize the assembly. Genome completeness was evaluated using BUSCO v5 (Simao et al, 2015) with the alveolata db10 (171 orthologs) and stramenopiles odb10 (100 orthologs) datasets (Table S1). Analyses were performed separately for haplotypes 1 and 2 as well as for the unplaced contigs. Telomere repeat units were examined using TIDK v.0.2.63 (Brown et al, 2025). The analysis was conducted with tidk explore, targeting the terminal 1% of each chromosome and considering repeat units between 5 and 12 base pairs in length, with a minimum of two consecutive repetitions. The canonical telomeric repeat sequence TTTAGGG (Fulneckova et al, 2013) was subsequently identified using tidk search. Visualization revealed a nearly complete assembly from telomere to telomere. Telomeric repeats were absent at one end of chromosomes 5 and 17 in both haplotype 1 and 2, as well as chromosome 14 in haplotype 2.

The annotation from the most recent assembly of reference strain Pv221_1 (available at https://doi.org/10.57745/MXJWZS) was transferred to the two haplotype assemblies of Pv1419_1 using liftoff v1.6.2 (Shumate and Salzberg, 2021). Additionally, an open reading frame (ORF) search was performed along the QTL detected in this strain to reveal single-exon coding sequences that were absent from the reference genome. ORFs with a minimum length of 200 pb were detected using r/ORFik v1.24 (Tjeldnes et al, 2021). Amino acid sequences were extracted and submitted to InterProScan to identify protein domains (Jones et al, 2014). Sequences displaying transposon-associated domains were discarded. The remaining protein sequences were predicted to be secreted by detecting signal peptides with SignalP v5.0 (Almagro Armenteros et al, 2019).

**Methods S2** Genotyping of the backcross population by amplicon length polymorphism

Specific primers were designed to amplify DNA segments encompassing three indels, thus generating different amplicon lengths between alleles: 430 vs 766 pb for the first marker, 240 vs 199 pb for the second, and 153 versus 119 pb for the third. PCR amplifications were performed in a 20 µL reaction volume consisting of 8.2 µL of nuclease-free water, 10 µL of Platinum II Hot-Start PCR Master Mix, 0.4 µL of each primer at a concentration of 10 µM, and 1 µL of DNA template. The reactions were performed in an Applied Biosystems VeritiPro Thermal Cycler with the following program: 120 s at 94°C for initial denaturation, followed by 40 cycles of denaturation (30 s at 94°C), annealing (15 s at 55 or 60°C depending on the primers) and extension (30 s at 68°C). PCR products were visualized by electrophoresis on agarose gels. Primer sequences and annealing temperatures are indicated in Table S2.

**Methods S3** Mapping, variant calling and filtration

We used a pipeline implemented in RattleSNP (https://rattlesnp.readthedocs.io/) to parallelize read mapping and variant calling. Mapping on the Pv221 1 primary haplotype assembly was done with bwa-mem v0.7.18 (Li, 2013). Bam files of dierent libraries of the same sample were merged using samtools v1.18 (Li et al, 2009). Variant calling was performed using GATK v4.2.6 (McKenna et al, 2010). PCR duplicate reads were removed with the command Picard MarkDuplicates. Genotypes were called using GATK HaplotypeCaller with default parameters. Variants in repetitive regions were filtered out with bedtools v2.30 (Quinlan and Hall, 2010), based on the repeat library built by Dussert et al (2019). High-confidence SNPs were retained using bcftools v1.17. Biallelic SNPs were the only sites considered. Sites with a depth twice as high as the average individual coverage were set to missing as they could be the result of false heterozygosity due to non-annotated transposable elements (TE) or copy number variation (CNV). SNPs with a depth inferior to 5 in a sample were set to missing. Finally, variants missing in more than 10% of the population were removed.

**References**:

Almagro Armenteros JJ, Tsirigos KD, Sønderby CK, Petersen TN, Winther O, Brunak S, von Heijne G, Nielsen H (2019) SignalP 5.0 improves signal peptide predictions using deep neural networks. Nat Biotechnol 37(4):420–423. [https://doi. org/10.1038/s41587-019-0036-z](https://doi.org/10.1038/s41587-019-0036-z)

Brown MR, Manuel Gonzalez de La Rosa P, Blaxter M (2025) tidk: a toolkit to rapidly identify telomeric repeats from

genomic datasets. Bioinformatics 41(2):btaf049.

<https://doi.org/10.1093/bioinformatics/btaf049>

Cheng H, Concepcion GT, Feng X, Zhang H, Li H (2021) Haplotype-resolved de novo assembly using phased assembly

graphs with hifiasm. Nat Methods 18(2):170–175. <https://doi.org/10.1038/s41592-020-01056-5>

Dussert Y, Mazet ID, Couture C, Gouzy J, Piron MC, Kuchly C, Bouchez O, Rispe C, Mestre P, Delmotte F (2019) A High-

Quality Grapevine Downy Mildew Genome Assembly Reveals Rapidly Evolving and Lineage-Specific Putative Host

Adaptation Genes. Genome Biology and Evolution 11(3):954–969. https://doi.org/10.1093/gbe/evz048

Dvorak E, Mazet ID, Couture C, Delmotte F, Foulongne-Oriol M (2025) Recombination landscape and karyotypic

variations revealed by linkage mapping in the grapevine downy mildew pathogen Plasmopara viticola. G3 Genes|Genomes|Genetics 15(1):jkae259. <https://doi.org/10.1093/g3journal/jkae259>

Fulneckova J, Sevcikova T, Fajkus J, Lukesova A, Lukes M, Vlcek Lang BF, Kim E, Elias M, Sykorova E (2013) A Broad Phylogenetic Survey Unveils the Diversity and Evolution of Telomeres in Eukaryotes. Genome Biology and Evolution 5(3):468–483. <https://doi.org/10.1093/gbe/evt019>

Huson DH, Beier S, Flade I, Gorska A, El-Hadidi M, Mitra S, Ruscheweyh HJ, Tappu R (2016) MEGAN Community

Edition - Exploration and Analysis of Large-Scale Microbiome Sequencing Data. PLOS Computational Biology 12(6):e1004957. <https://doi.org/10.1371/journal.pcbi.1004957>

Jones P, Binns D, Chang HY, Fraser M, Li W, McAnulla C, McWilliam H, Maslen J, Mitchell A, Nuka G, et al (2014)

InterProScan 5: genome-scale protein function classification. Bioinformatics 30(9):1236–1240. [https://doi.org/10.1093/ bioinformatics/btu031](https://doi.org/10.1093/bioinformatics/btu031)

Li H (2013) Aligning sequence reads, clone sequences and assembly contigs with BWA-MEM. https://doi.org/10.48550/ arXiv.1303.3997, URL http://arxiv.org/abs/1303.3997

Li H, Handsaker B, Wysoker A, Fennell T, Ruan J, Homer N, Marth G, Abecasis G, Durbin R, 1000 Genome Project Data Processing Subgroup (2009) The Sequence Alignment/Map format and SAMtools. Bioinformatics 25(16):2078–2079. https://doi.org/10.1093/bioinformatics/btp352

McKenna A, Hanna M, Banks E, Sivachenko A, Cibulskis K, Kernytsky A, Garimella K, Altshuler D, Gabriel S, Daly M, et al (2010) The Genome Analysis Toolkit: A MapReduce framework for analyzing next-generation DNA sequencing data. Genome Res 20(9):1297–1303. https://doi.org/10.1101/gr.107524.110

Minio A, Cochetel N, Vondras AM, Massonnet M, Cantu D (2022) Assembly of complete diploid-phased chromosomes from draft genome sequences. G3 Genes Genomes Genetics 12(8):jkac143. <https://doi.org/10.1093/g3journal/jkac143>

O’Leary NA, Wright MW, Brister JR, Ciufo S, Haddad D, McVeigh R, Rajput B, Robbertse B, Smith-White B, Ako-Adjei

D, et al (2016) Reference sequence (RefSeq) database at NCBI: current status, taxonomic expansion, and functional annotation. Nucleic Acids Research 44(D1):D733–D745. <https://doi.org/10.1093/nar/gkv1189>

Quinlan AR, Hall IM (2010) BEDTools: a flexible suite of utilities for comparing genomic features. Bioinformatics

26(6):841–842. <https://doi.org/10.1093/bioinformatics/btq033>

Shumate A, Salzberg SL (2021) Liftoff: accurate mapping of gene annotations. Bioinformatics 37(12):1639–1643.

<https://doi.org/10.1093/bioinformatics/btaa1016>

Simao FA, Waterhouse RM, Ioannidis P, Kriventseva EV, Zdobnov EM (2015) BUSCO: assessing genome assembly and annotation completeness with single-copy orthologs. Bioinformatics 31(19):3210–3212. [https://doi.org/10.1093/ bioinformatics/btv351](https://doi.org/10.1093/bioinformatics/btv351)

Tjeldnes H, Labun K, Torres Cleuren Y, Chyzy˙ nska K,´ Swirski M, Valen E (2021) ORFik: a comprehensive R toolkit for the analysis of translation. BMC Bioinformatics 22(1):336. <https://doi.org/10.1186/s12859-021-04254-w>

Voglmayr H, Greilhuber J (1998) Genome Size Determination in Peronosporales (Oomycota) by Feulgen Image

Analysis. Fungal Genetics and Biology 25(3):181–195. <https://doi.org/10.1006/fgbi.1998.1097>
