# Supplementary material for: Parallel adaptation and admixture drive the evolution of virulence in the grapevine downy mildew pathogen
Source: PLoS Pathog. 2026 Mar 10;22(3):e1014041. doi: 10.1371/journal.ppat.1014041 (PMC13048484; doi:10.1371/journal.ppat.1014041)
Supplement: Attachment — Submitted filename: Dvoraketal_ResponseToReviewers.pdf [file ppat.1014041.s020.pdf]

Response to reviewers' comments

Note: during revision, we spotted an error in the number of SNPs actually used in the population structure analyses, so we corrected it where it was mentioned (~~228,318~~ → 89,673).

Part II – Major Issues: Key Experiments Required for Acceptance

Reviewer #1:

1) The authors conclude that long stretches of homozygosity are indicative of recent selective sweeps. While I agree that this is a plausible indication, I believe additional analyses would strengthen this conclusion. For example, do strains that do not show virulence breakdown display a different pattern of homozygosity? One would expect this to be the case if the selective sweep was acting specifically on the virulent allele.

We thank the reviewer for their remarks. We improved Fig S6 to show runs of homozygosity in avirulent strains from the same regions than the virulent ones. We also extended the analysis to the entire chromosome. It shows that runs of homozygosity do not seem to follow any pattern in avirulents, while all virulent strains are homozygous around the AvrRpv12 locus. The updated figure is now Fig S7A (the blue box indicates the AvrRpv12 locus).

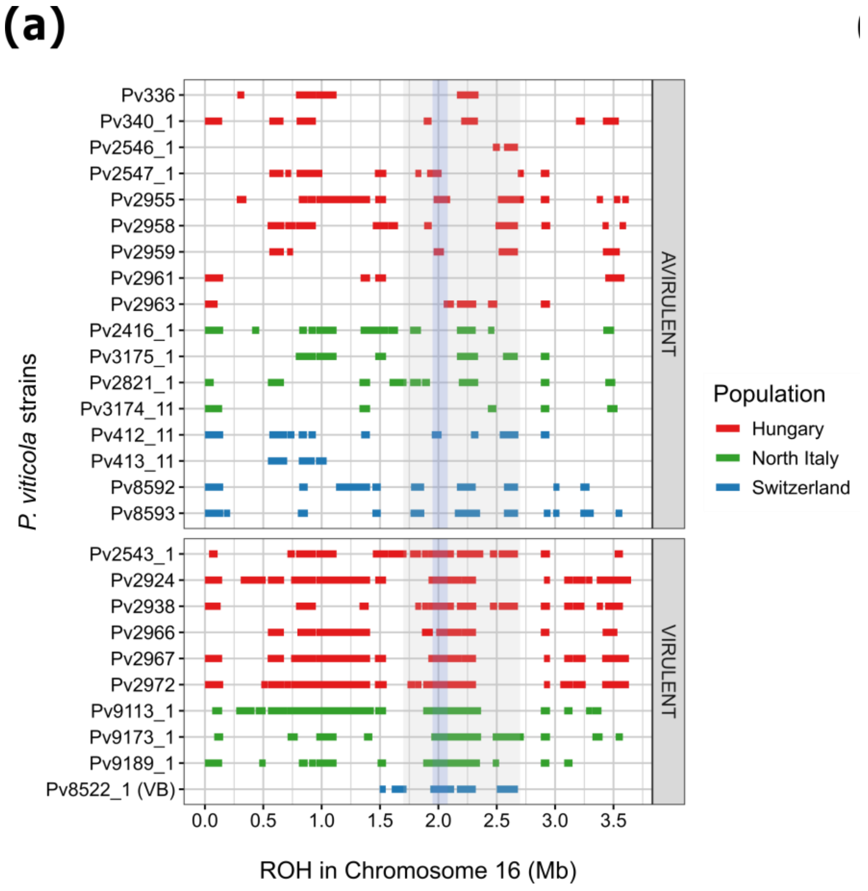

Additionally, the authors should consider more formal approaches to detect selective sweeps. For instance, using tools like isoRelate to identify identity-by-descent (IBD) segments could provide more robust evidence for recent sweeps.

We are aware that our ability to detect positive selection is limited by our small sample size (especially since the potentially selected haplotypes are different in each subpopulation). That being said, we used Selscan 2.0 to try to detect a selective sweep (https://doi.org/10.1093/bioinformatics/btae006). We calculated the cross-population Extended Haplotype Homozygosity (XP-EHH) (a statistic which detect SNPs approaching fixation in a population while remaining polymorphic in the other) and we applied it to compare the avirulent and virulent strains in the Hungarian group (n=15). The strongest signal of positive selection was found around AvrRpv12 in the virulent subpopulation. We added this figure as Fig S7B.

(b)

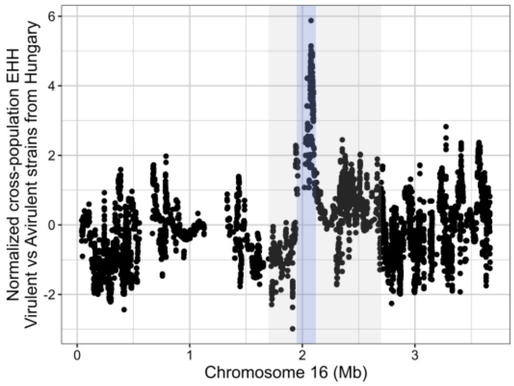

In addition, we followed the suggestion of Reviewer #1 and compared the proportion in IBD segments using r/isoRelate (https://doi.org/10.1371/journal.pgen.1007279). We observe a higher proportion of IBD in virulent strains compared to avirulent ones (both in Hungary and Italy). However, we are not sure of the reliability and precision of this type of analysis for such small sample sizes, so we decided not to include it in the paper.

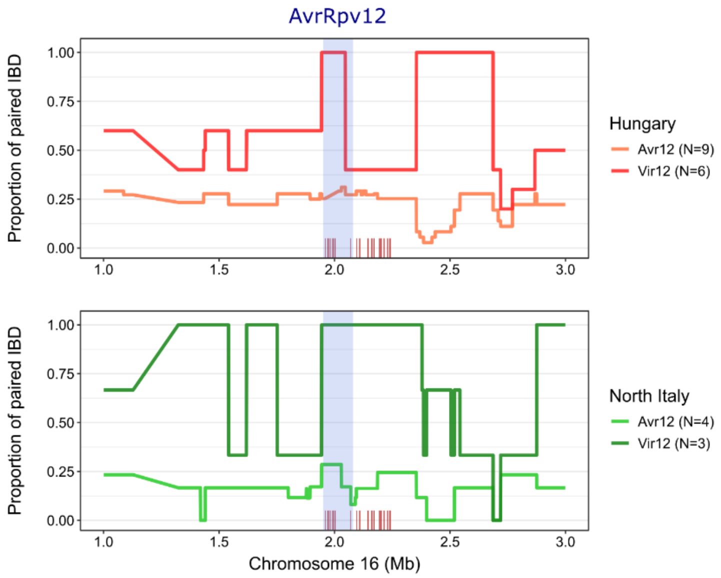

We modified the text accordingly. (line 252-262 in the Methods, line 347-351 in the Results, line 542-545 in the Discussion)

"In addition, we investigated signs of a selective sweep at the locus in virulent strains. The analyses were carried out at the chromosome scale by ordering and orienting contigs based on the linkage map. Runs of homozygosity were detected and visualized with r/detectRUNS v.0.9.6 (Biscarini et al 2019) using sliding windows of 15 SNPs. The parameters required to identify a segment were set to a minimum number of 20 SNPs, a maximum number of 1 heterozygous sites, a minimum length of 50 kb, and a maximum gap length of 100 kb. We also compared signals of positive selection in virulent and avirulent strains in the Hungarian group (n=15). To do so, we calculated the cross-population Extended Haplotype Homozygosity (XP-EHH), a statistic which detect SNPs approaching fixation in a population while remaining polymorphic in the other (Sabeti et al 2007). We used the method implemented in selscan v2.0.3 (Szpiech 2024) for unphased data, with default parameters except for a maximum gap length of 250,000 kb."

"Compared to avirulent strains from similar geographical origins, Rpv12-breaking strains consistently exhibited long runs of homozygosity around the deletions (Fig. S7A), which could be the result of a recent selective sweep. Besides, in our panel of Hungarian strains, the strongest signal of positive selection was observed around the locus in the virulent subpopulation (Fig S7B)."

"Moreover, Rpv12-breaking strains exhibited signs of a selective sweep at the locus, in line with the recent introduction of Rpv12-carrying varieties."

2. On a related note, the claim based on the admixture analysis could benefit from additional consideration and perhaps some discussion of the existing literature. For example, Fountain et al., 2021, which is cited throughout the discussion, did not detect any admixture between the American and European populations, but rather between the European and South African populations. In, other words, could the relatively limited number of non-European isolates in the current study be skewing the admixture analysis? A discussion of this limitation, and how it might affect the interpretation of the results, should be included.

As these concerns overlap with the issue raised by Reviewer #2, we answer both here:

We agree that the limited sampling outside Europe can bias the analysis and that we cannot exclude that the actual source population could be unsampled. We revised the Discussion to be less assertive and explicitly acknowledge this possibility, in line with the literature on biases in admixture analyses (lines 587-599).

"[...] Nevertheless, our limited sample size may bias the genetic clustering analyses. The admixture signal could be incorrectly attributed to the North American cluster because the actual source population may be insufficiently sampled or absent from the panel (Lawson et al 2018, Toyama et al 2020)."

To challenge our hypothesis of a North American origin, we ran qPCRs to amplify a candidate effector gene absent in the European genetic background but present in the virulent S10 haplotype. We tested 93 USA samples from previous works. We detected the gene in 43% of the samples (in different states and in the wild or cultivated compartments). While this

is not a definitive proof, this confirms that this gene is present in North American populations (where it seems subject to presence-absence variation). We included this result in the manuscript (see Methods line 263-275 and Results line 474-479).

We consider a North American origin plausible, as this region is the centre of origin of *P. viticola* and all other populations studied worldwide derive from the European population, as shown by Fontaine et al 2021. It is true that this study did not detect any admixture between the American and European clusters, but the sampling differed widely compared to the present study. In Fontaine et al 2021, samples were collected on susceptible varieties in ~2008-2014. In the present study, admixed strains were found only on Rpv10 plants, and only in the precise region where Rpv10 breakdown was first reported. The strain PV1419 seems to be an early generation hybrid and was found in 2012, suggesting that the secondary introduction of the pathogen into Europe was very recent at the time – making it unlikely for Fontaine et al to collect an admixed isolate by chance.

Reviewer #2:

Were all the strains in Figure 4A phenotyped on Rpv10 or only the ones identified as resistance breaking?

We thank the reviewer for their remarks. All strains were phenotyped on Rpv10, except the American ones (and the Chinese sequence retrieved from SRA). We deliberately do not import living *P. viticola* from America, precisely because of the risks of leak into the environment, so the DNA was extracted in Canada/USA. In any case, we do not think that these three North American strains were necessarily virulent on Rpv10.

Is the introgressed haplotype in the isolates that have overcome Rpv10 identical to the sequenced isolates from North America or is the ADMIXTURE analysis clustering them because the actual source population was not sampled? For example, Fontaine et al. 2021 genotyped isolates from China and the authors mention that the Rpv10 resistance was sourced from East Asia. Could it be that the introgression was from an unsampled V. aestivalis population? I don't question the introgression but I wonder about the inference that the US is the source given the limited sampling of other populations and their close relationships. The authors sometimes qualify the inference, for example "extra-European population" on line 414, but the discussion is more definitive about a secondary introduction from North America.

As these questions overlap with the issue raised by Reviewer #1, we addressed these concerns in our response above to Reviewer #1's second remark.

Reviewer #3:

(No Response)

Part III – Minor Issues: Editorial and Data Presentation Modifications
Please use this section for editorial suggestions as well as relatively minor modifications of existing data that would enhance clarity.

Reviewer #1:
It personally took me a while to understand why there are genetic maps for both parental isolates separately. This is probably very obvious to someone working with a diploid species, but for those of us who don't, a little explanation would certainly help with understanding the manuscript.

Also, at the beginning of the Results section (around Line 225), it might be good to restate what kind of population these are.

As suggested, we added some details in the Methods section and at the beginning of the Results section. (line 167-168, line 278-282)

"We employed a pseudo-testcross mapping strategy, which is suitable for a self-incompatible and highly heterozygous species such as P. viticola."

"Two biparental F1 populations were derived from the cross of P. viticola strains [...] Due to the pseudo-testcross mapping strategy, two parental linkage maps were built for each cross and QTLs could be detected when a parent strain was heterozygous at the locus."

Line-specific comments:

Line 136: Please include a reference to the supplementary figure showing the scale.

We referenced Fig S1. (line 152)

Line 155: A bit more detail would be helpful. What was the command used to run the analysis—was it scanone? Same question for composite interval mapping.

We detailed the use of r/qtl functions scanone and cim. (line 172, 178)

Line 216: What subset of the total number of SNPs was considered "fixed" in this analysis?

We added the number of SNPs retained in the Method section. (line 237-240)

"We used only a subset of SNPs that were fixed in each "ancestral" population (homozygous reference allele in the subset of European strains not showing admixture with the North American cluster, homozygous alternate allele in the North American population). Thus, 328,439 potential SNPs were suitable for the analysis, including 33,832 in chromosome 16, where the region of interest was located. The set of variants in this chromosome was filtered as described above, and then thinned with PLINK to achieve a density of about 1 SNP/kb, retaining 3876 SNPs."

Line 217: Which strains were defined as pure "European" ancestry, given that Admixture identifies two ancestries for Europe at K=3, correct?

We clarified that we referred to European strains without admixture from a likely North American source (line 235-236).

“As this approach required variant phasing, we used only a subset of SNPs that were fixed in each "ancestral" population (homozygous reference allele in the subset of European strains not showing admixture with the North American cluster, homozygous alternate allele in the North American population).”

Line 245: High levels of sporulation tended to be associated with light necrosis surrounding sporulation spots (). Where is this data shown?

We added a reference to figure 1A where sporulation and necrosis scores are visualized together. (line 301)

Line 256: Which reference genome was used?
Line 257: What is the gene name?

We used the 2nd version of the reference genome of strain Pv221 (Paineau 2024). The sequence was badly annotated in the annotation of the 2nd version (Paineau 2024), so we opted to present the sequence with the “old name”. PVIT_0017314 was the gene name in the annotation of the 1st version (Dussert 2019).
We reformulated the sentence to make this clearer. (line 312)

Line 296: What is the sporulation phenotype of this isolate?

We added this information. (line 354-355)

“This strain induced poor sporulation on all cultivars, possibly due to its karyotypic anomaly. Interestingly, it did not trigger any necrosis on Rpv12 plants (average necrosis score = 0).”

Line 382: What is the CV error? Where is this data shown?

We added a supplementary figure showing the cross-validation (CV) error calculated by ADMIXTURE depending on the number of cluster (Fig S8).

Data availability:
Maybe you could extend the data availability statement, saying that not only genome and annotation are available through the provided link, but also phenotypes and map data.

The statement was completed as suggested.

Supplementary Methods and Figures:

Methods S1: This section is lacking a bit of technical depth. How was the hifiasm program run (with what specifications)? What was the threshold for keeping a contig? With what parameters was hifiasm run? How was the genetic map used to assemble the contigs? Was short-read data used to phase the genomes?

The description of the assembly pipeline has been expanded to include these details. Details of the evaluation criteria have been added to Methods S1 as well as the final selected HiFiam parameters:

"To generate the most contiguous and high-quality draft genome assembly and determine the optimal settings in HiFiasm, multiple parameter combinations were tested, generating a total of 252 diploid assemblies. All assemblies were evaluated for haplotype balance, length and overall quality. Each haplotype was expected to have a cumulative length between 100 and 115 Mbp, with a difference of less than 10 Mbp between the two haplotypes. Assembly fragmentation was assessed based on contig length distribution, with high-quality assemblies characterized by a longest contig exceeding 7.5 Mbp and a high proportion of large contigs. To assess assembly completeness and uniformity, NG-values (NG0–NG99) were calculated for both haplotypes using an expected genome size of 115 Mbp (Voglmayr and Greilhuber, 1998). The full range of NG-values was compared between haplotypes to verify their homogeneity, ensuring consistent contiguity and completeness across the genome and avoiding cases where one haplotype displayed substantially higher NG-values than the other."

"HiFi reads corresponding to contaminant contigs were removed, and HiFiasm was rerun using the cleaned dataset. The resulting assemblies were evaluated according to the quality metrics described above. Genetic markers were then mapped onto the best assemblies to assess completeness and guide the final selection. The assembly generated with parameters a = 2, k = 61, w = 71, f = 25, r = 5, s = 0.6, D = 10, N = 75, n = 10, and m = 500,000 showed the highest completeness and overall quality (Table 1) and was selected for the second step of the assembly."

How was the genetic map used to assemble the contigs?

The diploid genome was assembled from PacBio HiFi reads with HiFiasm, which resolves heterozygous regions into distinct haplotype paths, and scaffolded into chromosome-scale pseudomolecules with HaploSync using sequence alignments and the genetic map.

The genetic map was used during two later stages: first, in the contaminant-filtering step of the HiFi reads, and second, during scaffolding with HaploSync. In this step, the genetic map was used in HaploSplit to guide the chromosome assignment and orientation of contigs based on the order and consistency of genetic markers, ensuring accurate and marker-supported scaffolding of both haplotypes (Minio et al., 2022).

This has been clarified in the Methods S1:

"Next, scaffolded, haplotype-resolved, chromosome-scale pseudomolecules were constructed for both haplotypes (Hap1 and Hap2; Table 1) using HaploSync tool suite v1.0 (Minio et al., 2022) and with the P. viticola genetic map (Dvorak et al., 2025) as external evidence. The genetic map was used in HaploSplit to guide the chromosome assignment and orientation of contigs based on the order and consistency of genetic markers, ensuring accurate and marker-supported scaffolding of both haplotypes (Minio et al., 2022)."

Was short-read data used to phase the genomes?

No short-read data were required to resolve both haplotypes of the assembly, as HaploSync leverages sequence alignments and genetic map information to independently reconstruct

each haplotype. In this section, we initially described the assembly as “phased,” but we agree that this term is not accurate and we have replaced it with the term “haplotype-resolved” (like in the main text). However, during our later analyses, we used markers from the genetic map to verify that the two haplotypes were correctly phased (no “haplotype switching”) in the chromosome 16, in which the S-AvrRpv10 locus was found.

Methods S2: Please indicate where the primer sequences can be found.

Primer sequences are indicated in table S2.

Methods S3: How were repetitive regions defined? Did you perform repeat masking with a repeat library?

Repetitive regions were defined based on the repeat library built by Dussert et al 2019. We added this information.

Figure S6: What does the yellow box indicate?

We modified this figure according to Reviewer #1’s major concern above.

Reviewer #2:
1. I advise replacing “pure” in reference to European and North American samples (line 403). Given that the European samples are descendants of the North American population, I’m not sure this is an appropriate term. And it is problematic in the larger context of population genetics, so best to not use it and instead refer to SNPs associated with ADMIXTURE clusters.

We agree with the reviewer and modified the text accordingly. (line 463-465)
“Ancestral segments were defined using a subset of 3876 fixed SNPs between the North American population and European strains for which no American admixture was detected”

2. That the RXLR motif can take alternative forms, particularly in downy mildews: RXLK, RVRN, QXLR, GKLR, etc. For the proteins that have the dEER motif, do these have an RXLR-like motif? This information would be useful for computational identification of cytoplasmic effectors in other Plasmopara, especially if those that don’t have dEER or LWY.

We did not identify any additional motifs in these candidate effectors.

3. The three North American isolates sequenced were identified as belonging to “clade aestivalis, the only species present worldwide” (line 191). The reader must infer, or look at Fontaine et al. 2021, to determine that the strains being examined from Europe are also clade aestivalis.

We clarified the text as suggested. (line 209-210)

“We sequenced 41 new strains, all belonging to P. viticola f. sp. aestivalis, the only species present worldwide.”

4. Tandem is used as a noun but I typically think of it as an adjective – tandem array of genes or tandem duplicate genes.

We corrected the sentences as suggested. (line 95, 291)

5. I suggest point mutations or nucleotide substitutions instead of "punctual variants".

We corrected the sentence as suggested. (line 409, 413)

Reviewer #3:
L104-107: Although it is mentioned later in the manuscript, stating the genotypes of the cPv44_ 1 and Pv1419_1 strains here when mentioning the backcross population would help make the objective/rationale for this experiment clearer.

We thank the reviewer for their remarks. We rephrased the text to make the objective clearer. (line 119-121)

"In addition, a backcross population was produced in order to further test the dominance of virulence towards Rpv10. The virulent strain cPv44_1 from the 412x1419 population was backcrossed to the parent Pv1419_1."

-In Methods S1, it is stated "The ten most promising assemblies were then screened for contamination.". What criteria were used to select these ten assemblies?

The description of the assembly pipeline has been expanded to clarify this point. To select the best assemblies, multiple combinations of HiFiasm parameters were tested, and the resulting assemblies were evaluated using descriptive statistics assessing haplotype balance, total genome length, and overall assembly quality. Details of these evaluation criteria have been added to Methods S1:

"To generate the most contiguous and high-quality draft genome assembly and determine the optimal settings in HiFiasm, multiple parameter combinations were tested, generating a total of 252 diploid assemblies. All assemblies were evaluated for haplotype balance, length and overall quality. Each haplotype was expected to have a cumulative length between 100 and 115 Mbp, with a difference of less than 10 Mbp between the two haplotypes. Assembly fragmentation was assessed based on contig length distribution, with high-quality assemblies characterized by a longest contig exceeding 7.5 Mbp and a high proportion of large contigs. To assess assembly completeness and uniformity, NG-values (NG0–NG99) were calculated for both haplotypes using an expected genome size of 115 Mbp (Voglmayr and Greilhuber, 1998). The full range of NG-values was compared between haplotypes to verify their homogeneity, ensuring consistent contiguity and completeness across the genome and avoiding cases where one haplotype displayed substantially higher NG-values than the other. Based on these descriptive statistics, the ten most promising assemblies were screened for contamination."

Fig. S4 – The methods used in this analysis are not presented in the manuscript.

We added the methods to the main text. (line 245-248)

"A maximum-likelihood phylogenetic tree was build using protein sequences of putative effectors in the locus to compare it to the physical distances between genes. We used the method implemented in MEGA11 with an initial Neighbor-Joining tree, 1000 bootstrap replicates and otherwise default parameters (Jones-Taylor-Thornton substitution model, uniform rates among sites, fast SPR heuristic method)."

Fig. S5 – Methods also missing for the read depth analysis.

We added the methods to the main text. (line 249-251)

"The presence-absence of genes was inferred from the depth of coverage at the locus. Per-base read depth was obtained with samtools v1.18 (Li et al, 2009). The mean coverage value was calculated on 5-kb windows and normalized based on the genome-wide average for each strain, assuming diploidy."

L394-396: Could the lower level of admixture observed in the Rpv10-breaking strains besides Pv1419_1 be caused by recurrent backcrossing to the Upper Rhine Plain population? There also seems to be decent separation between these three Rpv10-breaking strains and the rest of the cluster in the lower-left of the PCA plot.

This is indeed our hypothesis, as the other Rpv10-breaking strains were collected more recently. For example, the Pv7219 strain shows the lowest "American" admixture (17%) but it is largely concentrated on chr16, where the virulence locus is located.
We added a sentence to state it explicitly. (line 459-460)

"The lower admixture levels observed in strains collected more recently (between 2020 and 2023) could be the result of recurrent backcrossing with the local population."
