# Supplementary material for: Parallel adaptation and admixture drive the evolution of virulence in the grapevine downy mildew pathogen
Source: PLoS Pathog. 2026 Mar 10;22(3):e1014041. doi: 10.1371/journal.ppat.1014041 (PMC13048484; doi:10.1371/journal.ppat.1014041)
Supplement: Attachment — Submitted filename: Dvoraketal_ResponseToComments_2.pdf [file ppat.1014041.s021.pdf]

Response to editor comments

We would like to thank the editor and the reviewers for their final assessment and for taking the time to review this revised version of our manuscript. Please find our detailed responses below and the corresponding corrections implemented in the revised version of the manuscript.

Additional Editor Comments:

The reviewers all agree that all concerns were raised satisfactorily. I have just a few minor points before the manuscript can be finally accepted. These small text and formatting-related issues should be addressed.

The abstract should just be one paragraph, so please remove line breaks.
We modified the abstract accordingly.

Please revise and improve text in Author Summary including:
Line 37 insert "…the resistance genes…"
Line 39 Enriched in stead of rich
Line 40 something is missing "…One??"

Intro:
Line 92: Please change: "Thanks to recent advances in the sequencing and annotation of the genome of P. viticola, the first Avr locus was identified….."

Methods:'
Line 250: "….in 5 kb windowns…."

We corrected the text as suggested. (lines 36-38, line 89-91, line 248).

Results:
Line 289: is "credible interval" correct here? I think the term mainly used in Bayesian analyses. Shouldn't it be "confidence interval" here? And in Figure legend 1 and 2 and supplementary figures? Please check which term is correct according to the analysis used.

We indeed opted to determine Bayesian credible intervals, using the bayesint function implemented in r/qtl (see line 173). Therefore, we have retained the term "credible interval" in the result section to remain consistent with our analysis.

Line 460: Please insert: "… recurrent backcrossing with the local P. viticola population"

We completed the text as suggested. (line 457).

Methods S1-S3: I recommend joining these files into one supplementary file "Supplementary Methods"
Please insert title of manuscript and author names on first page of the document.

We merged the documents as suggested and indicated the manuscript and authors in the first page. (file now titled “Supplementary Methods”).
